# Angioleiomyoma of the nasal septum

**DOI:** 10.1590/S1808-86942010000500027

**Published:** 2015-10-22

**Authors:** Carlos Roberto Ribeiro NavarroJúnior, Adriano Santana Fonseca, José Rodrigo Lordello de Mattos, Nilvano Alves de Andrade

**Affiliations:** 1 MD. Resident Physician in ENT and HNS - Santa Casa de Misericórdia da Bahia; 2 MD. ENT and Maxillo-facial surgeon. Preceptor at the ENT Residency Program - Santa Casa de Misericórdia da Bahia; 3 MD. ENT and Maxillo-facial surgeon. Preceptor at the ENT Residency Program - Santa Casa de Misericórdia da Bahia; 4 PhD in Surgery - USP, Head of the ENT Residency Program - Santa Casa de Misericórdia de Bahia

**Keywords:** leiomyoma, nose neoplasms, nasal septum.

## INTRODUCTION

Leiomyoma is a benign smooth muscle tumor, more commonly found in the uterus (95%), skin (3%), nutritional and gastrointestinal tracts (1.5%)^1^. It was initially described in the nasal cavity by Maesaka et al. in 196^6^,^2^.

The goal was to describe a case with clinical manifestations and histopathology findings of angioleiomyoma of the nasal septum, a rare benign neoplasia which represents less than 1% of all the leiomyomas in the human body^3^.

## CASE PRESENTATION

**Figure 1:**
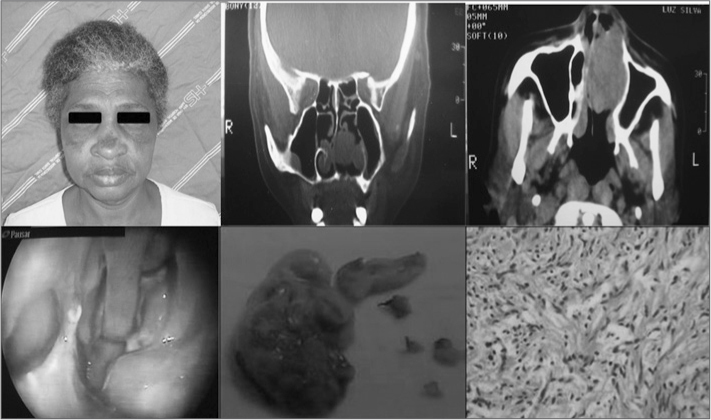
Set of photographs from this patient, CT scan, late post-op, macroscopy and microscopy of the lesion.

M.F.L.S., 62 years, female, Africandescendant, she came to our ENT service complaining of a tumor in her left nasal cavity with six years of evolution. In the three initial years it had a progressive growth, associated with low volume epistaxis episodes. After such period, she developed nasal obstruction on the left side and facial pain. Upon physical exam and fibroscopy we noticed a brown, smooth, pedicled lesion on the left-side septum, well outlined, measuring approximately 4 × 2cm, completely occluding the left nasal cavity and pushing the nasal septum. CT scan of the paranasal sinuses showed a well-outlined soft tissue mass, pushing the septum and the lateral wall. Biopsy reported leiomyoma. Later on, the tumor was endoscopically resected, with a 1cm margin, considered adequate according to anatomical and pathological criteria. Microscopy showed polypoid fragments, coated by a single layer of cylindrical hair cells, typical pseudostratified, showing in the stroma, typical leiomyoma bundles around the thick walls of vessels. (Fig. 1)

## DISCUSSION AND FINAL REMARKS

This is a slow growth tumor. The most common symptoms are: nasal obstruction, epistaxis, facial pain and headaches. He most frequent treatment for nasal septum angioleiomyoma is endoscopic resection with macroscopic margin, and this was the treatment option for this case - excision with macroscopic and microscopic free margins. Vascular leiomyomas are bundles of smooth muscle cells, relatively organized, and permeated by thick wall vessels^4^.

The nasal septum vascular leiomyoma is an extremely rare tumor, of uncertain origin^5^. Resection is the procedure of choice and it bears a high cure rate. The endoscopic procedure is a good option for small to moderate size tumors^6^.
